# C-856-02. Impact of Posttraumatic Stress and Depression Symptom Severity on Functional Outcomes in Adult Burns

**DOI:** 10.1093/jbcr/irag033.094

**Published:** 2026-04-06

**Authors:** Sarah A Stoycos, Kara McMullen, Shelley A Wiechman, Kimberly Roaten, Caitlin M Orton, Jeffrey C Schneider, Mladen Nisavic, Lewis E Kazis, Haig A Yenikomshian

**Affiliations:** Keck School of Medicine, University of Southern California, Los Angeles, CA; Department of Rehabilitation Medicine, University of Washington, Seattle, WA; University of Washington Harborview Medical Center, Seattle, WA; Department of Psychiatry, University of Texas Southwestern Medical Center, Dallas, TX; Department of Surgery, University of Washington Medicine Regional Burn Center, Harborview Medical Center, Seattle, WA; Spaulding Rehabilitation Hospital, Mass General Brigham, Harvard Medical School, Boston, MA; Harvard Medical School, Boston, MA; Boston University School of Public Health, Boston, MA; Division of Plastic and Reconstructive Surgery, Keck School of Medicine, University of Southern California, Los Angeles, CA

## Abstract

**Introduction:**

Posttraumatic stress (PTS) and depression (Dep) symptoms after burn injury are prevalent, impacting about one-third of survivors. Without intervention, PTS and Dep symptoms are unlikely to remit and are likely to impact functioning. This study examined the association of PTS (assessed using PCL-C) and Dep (assessed using PROMIS-29) symptoms at 6 months on physical function, ability to participate in social roles, pain intensity (all assessed using PROMIS-29), satisfaction with life (SWL), and community integration (CI) at 12 months, while controlling for sex, age, ethnicity, and burn injury characteristics (total body surface area (TBSA) burned, ventilation days).

**Methods:**

Utilizing data from a national burn outcome database, hierarchical linear regression models for each outcome were conducted to identify the unique contribution of PTS and Dep symptoms. Models were assessed for multicollinearity. Delta R2 statistics considered the additive effects of PTS and Dep symptoms [block 3] beyond demographic [block 1] and injury [block 2] characteristics.

**Results:**

Data from 965 participants (2015-2022) were included. Across models, PTS and Dep symptoms at 6 months post-discharge predicted poorer outcomes at 12-months, including lower CI (PTS: B = –.04, *p*<.001; Dep: B = –.07, *p*<.001), SWL (PTS: B = –.20, *p*<.001; Dep: B = –.31, *p*<.001), and social role participation (PTS: B = –.032, *p*<.001; Dep: B = –.52, *p*<.001), worse pain (PTS: B = –.077, *p*<.001; Dep: B = –.11, *p*<.001), and worse physical function (PTS: B = –.22, *p*<.001; Dep: B = –.25, *p*<.001). Across all outcomes, PTS symptoms accounted for an additional 6.1-14.7% of variance, with models across outcomes accounting for 21.8% (pain)-35.1% (SWL) of the variance. Across all outcomes, Dep symptoms accounted for an additional 5.3-21.1% of variance, with models across outcomes accounting for 25.7% (CI)-38.4% (social role participation) of the variance. In both PTS and Dep models, Hispanic participants had lower CI and SWL; older individuals had worse social role participation, physical function, and pain; females had worse pain; and increased ventilator days were associated with worse social role participation, physical function, and pain. TBSA did not meaningfully contribute to any model.

**Conclusions:**

PTS and Dep symptoms add psychological burden, contribute to long term physical suffering, and poorer quality of life. Psychological symptom burden, not burn size, was predictive of long-term functioning. It is important to consider PTS and Dep symptoms when studying predictors of physical function and quality of life in burn survivors.

**Applicability of Research to Practice:**

Integrating screening and early intervention for PTS, Dep, and pain may be critical to improving both psychosocial and physical recovery trajectories. Future research should examine whether early referral to peer support programs or family interventions for Hispanic and older burn survivors may address health disparities in post-burn adjustment.

**Funding for the study:**

This work was supported by NIDILRR (grant 90DPBU0007) and the National Institute of Mental Health (1K23MH141296-01). The contents do not necessarily represent the policy of NIDILRR, NIMH, ACL, or HHS, and endorsement by the Federal Government should not be assumed.

